# A Reliability Assessment Method for Complex Systems Based on Non-Homogeneous Markov Processes

**DOI:** 10.3390/s24113446

**Published:** 2024-05-27

**Authors:** Xiaolei Pan, Hongxiao Chen, Ao Shen, Dongdong Zhao, Xiaoyan Su

**Affiliations:** 1 College of Automation Engineering, Shanghai University of Electric Power, Shanghai 200090, China; chacnex@mail.shiep.edu.cn (H.C.); shenao8023@mail.shiep.edu.cn (A.S.); zhaodongdong@mail.shiep.edu.cn (D.Z.); suxiaoyan@shiep.edu.cn (X.S.); 2 Shanghai Key Laboratory of Power Station Automation Technology, Shanghai 200090, China

**Keywords:** reliability assessment, complex systems, curse of dimensionality, non-homogeneous Markov processes

## Abstract

The Markov method is a common reliability assessment method. It is often used to describe the dynamic characteristics of a system, such as its repairability, fault sequence and multiple degradation states. However, the “curse of dimensionality”, which refers to the exponential growth of the system state space with the increase in system complexity, presents a challenge to reliability assessments for complex systems based on the Markov method. In response to this challenge, a novel reliability assessment method for complex systems based on non-homogeneous Markov processes is proposed. This method entails the decomposition of a complex system into multilevel subsystems, each with a relatively small state space, in accordance with the system function. The homogeneous Markov model or the non-homogeneous Markov model is established for each subsystem/system from bottom to top. In order to utilize the outcomes of the lower-level subsystem models as inputs to the upper-level subsystem model, an algorithm is proposed for converting the unavailability curve of a subsystem into its corresponding 2×2 dynamic state transition probability matrix (STPM). The STPM is then employed as an input to the upper-level system’s non-homogeneous Markov model. A case study is presented using the reliability assessment of the Reactor Protection System (RPS) based on the proposed method, which is then compared with the models based on the other two contrast methods. This comparison verifies the effectiveness and accuracy of the proposed method.

## 1. Introduction

Reliability assessment is a crucial undertaking for complex systems such as nuclear power plants and aerospace systems. The outcomes of reliability assessments assist designers in enhancing their design processes, thereby improving the overall reliability of the system. Additionally, they assist operational and maintenance personnel in acquiring a comprehensive understanding of the associated risks, which provides a foundation for the implementation of informed risk management and decision-making [1].

There are dozens of methods commonly employed for system reliability assessment, including fault tree analysis (FTA), event tree analysis (ETA), the Markov method, dynamic flowgraph methodology (DFM), dynamic Bayesian, Monte Carlo simulation (MCS), and so on [2,3,4]. These reliability assessment methods are categorized as static and dynamic, depending on whether the dynamic characteristics of the system can be considered [5]. FTA and ETA are the typical static assessment methods that are often employed for the reliability assessments for complex systems, particularly in the field of nuclear power systems [6]. Although these methods are relatively straightforward and easy to implement, they are unable to express the dynamic characteristics of a system, which results in conservative outcomes [7,8]. The Markov method, DFM, dynamic Bayesian methods, and MCS are recognized as dynamic reliability assessment methods that offer certain advantages in presenting the dynamic characteristics of a system. However, each of these methods has its inherent limitations that limit their applications in practical engineering. Graphical dynamic reliability assessment methods, such as DFM and dynamic Bayesian, are often applied to system reliability assessments involving time series. The disadvantages of these methods are that the corresponding inference algorithm is required, and that the models are difficult to solve [9]. MCS is noted for their powerful ability to express dynamic characteristics, although it is computationally intensive and present significant difficulties in model validation [10,11].

The Markov method is regarded as a highly promising approach to reliability assessment [9]. It is frequently utilized to describe the dynamic characteristics of systems, such as reparability, timing, and multiple states for degradation process [12,13]. Numerous scholars have conducted relevant research in this area. For instance, Hellmich developed continuous-time Markov models to assess the reliability of two-train standby safety systems that consider organizing repair and testing [14]. Rajeevan discretized the degradation process into multiple states and employed the Markov method to complete the reliability modeling and analysis of repairable wind power systems [15]. Bessman used the Weibull distribution to describe the degradation process of components and established a non-homogeneous Markov model for the system [16]. The Markov method has been widely employed in reliability assessment.

One of the drawbacks of the Markov method is the “curse of dimensionality” problem, which weakens its applicability for reliability assessments of complex systems in practice [17,18,19]. The “curse of dimensionality” refers to the exponential explosive growth of the system state space and the scale of the Markov model with the number of system components [20]. The main research on the “curse of dimensionality” in reliability assessments based on the Markov method may be broadly divided into two categories: (1) The integration of other methodologies: In practice, Markov methods often are used in conjunction with other methods in reliability assessments of complex systems [21,22]. For instance, Abdulmunem employed a continuous-time Markov method in conjunction with the fault tree method to address the reliability modeling of complex systems [23]. The Markov method was used to model the small-scale subsystems, while the remainder of the system was modeled using the fault tree method. (2) The reduction of the scale of the Markov model: Son developed a systematic procedure for obtaining rate and transition matrices that optimally describe the dynamics of aggregated superstates formed by combining (clustering or lumping) microstates [24]. Liang proposed a method for simplification in continuous-time Markov state-based models for reliability assessments of complex safety systems and applied it to the reactor protection system (RPS) in nuclear power plants [19]. Ulla and Chen developed some practical methods to simplify Markov chains by removing low-equilibrium occupancy states with lower occupancy probabilities to reduce the scale of the Markov models [18,25,26]. Although significant achievements have been made in the research of the Markov method and its applications, the “curse of dimensionality” problem associated with the application of the Markov method has not yet been effectively resolved.

To address the aforementioned issue, a reliability assessment method based on non-homogeneous Markov processes is proposed in this paper. The degradation process is described by non-exponential distribution to enhance the accuracy of the model. Furthermore, the complex system is decomposed into multilevel subsystems according to the system function. The scales of the subsystems are controlled, and the homogeneous Markov models or non-homogeneous Markov models are established for each subsystem from bottom to top.

The main contributions of this paper are as follows:

(1) A reliability assessment method for complex systems based on non-homogeneous Markov processes is proposed. The effectiveness and accuracy of the proposed method are verified by the case study.
(2) An algorithm is proposed for converting the unavailability curve of a subsystem into its corresponding 2×2 dynamic state-transition probability matrix (STPM), which is then used as an input to update the dynamic STPM of the upper-level system’s non-homogeneous Markov model.
(3) Based on the proposed method, the reliability assessment of an RPS system of a nuclear power plant is completed. The unreliability of the system and two importance metrics, namely, the Risk Achievement Worth (RAW) and the Risk Reduction Worth (RRW), of each component were analyzed. The weaknesses of the system were then summarized. The outcomes of the reliability assessment will assist designers in enhancing their design processes and provide a foundation for the implementation of informed risk management and decision-making.

The reminder of this paper is arranged as follows: Section 2 provides the preliminary knowledge of this paper. In Section 3, the proposed reliability assessment method for complex systems based on a non-homogeneous Markov process is detailed. Section 4 introduces a case study: three reliability assessment models of the reactor protection system (RPS) are constructed using the proposed method and other two contrast methods to validate the effectiveness and accuracy of the proposed method. A conclusion and an outlook for future research are presented in Section 5.

## 2. Preliminaries

### 2.1. Markov Process

Markov processes are employed to study the random mutual transitions between the states of a system. If the probability of transitioning from one state to another is solely dependent on the current state and not on the previous state, then the process is referred to as a Markov process. The Markov process can be expressed by Equation (1), assuming that *S* is the set of states of the system.

(1) $$P\left\{X(t_{n})=x_{n}\left|X(t_{1})=x_{1},X(t_{2})=x_{2},\cdots X(t_{n-1})=x_{n-1}\right.\right\}=P\left\{X(t_{n})=x_{n}\left|X(t_{n-1})=x_{n-1}\right.\right\}$$

where $t_{1}<t_{2}<\cdots <t_{n}$; $X(t_{i})=x_{i}$ denotes that the system is in the state of $x_{i}$ at $t_{i}$, and $x_{i}\in S$.

A Markov process is considered homogeneous if the state transition is independent of the current state’s time, i.e.,

(2) $$P\left\{X(t+\Delta t)=k\left|X(t)=i\right.\right\}=P\left\{X(\Delta t)=j\left|X(0)=i\right.\right\}=p_{ij}(\Delta t)$$

where j,k∈S,Δt≥0; $p_{ij}(\Delta t)$ denotes the transition probability of the system moving from state *i* to *j* within time Δt and is only related to the size of the time interval Δt.

### 2.2. Reliability Assessment Based on the Markov Process

The International Electrotechnical Commission (IEC) has issued the IEC 61508 standard, which classifies the safety function operation of a component or system into three categories: low-demand mode, high-demand mode, and continuous mode [27]. In low-demand mode, the reliability measures are based on the average probability of state transition, depicted by a discrete distribution such as a Bernoulli distribution. For the cases of high-demand mode and continuous mode, the reliability measures are based on the average state transition frequency per hour, described by a continuous distribution. If all state transitions for high-demand mode and continuous mode follow exponential distributions, i.e., their transition rates are constant, the system state transition process is a homogeneous Markov process. Otherwise, it is a non-homogeneous Markov process.

#### 2.2.1. Reliability Assessment Based on the Discrete-Time Homogeneous Markov Process

The basic idea of a reliability assessment based on the discrete-time homogeneous Markov method is to discretize the mission time of the system into equal intervals and to observe the state transition of the system at each discrete time point. The interval between two adjacent time points is Δt, and if the interval is sufficiently small, it is assumed that at most a single state transition may occur for the system within a time period of Δt. The transition probability of the system from state *i* to state *j* can be expressed as follows:

(3) $$p_{ij}(\Delta t)=P\left\{X(t+\Delta t)=j\left|X(t)=i\right.\right\}$$

where i,j∈S.

In the absence of consideration of the low-demand mode, the STPM Λ, which represents the process of system state transitions within a Δt, can be expressed by Equation (4). The scale of the matrix Λ increases exponentially with the number of system components.

(4) $$\Lambda =\left|\begin{array}{cccc} p_{11}(\Delta t) & p_{12}(\Delta t) & \cdots & p_{1N}(\Delta t)\\ p_{21}(\Delta t) & p_{22}(\Delta t) & \cdots & p_{2N}(\Delta t)\\ \cdots & \cdots & \cdots & \cdots \\ p_{N1}(\Delta t) & p_{N2}(\Delta t) & \cdots & p_{NN}(\Delta t) \end{array}\right|$$

In consideration of the low-demand modes, such as the cold standby of a component, the system state moves from state 1 to state 2 when the cold standby component is successfully started. Otherwise, it moves to state 3. The probability of failure in starting the cold standby component is denoted by *q*, and the matrix Λ can be expressed by Equation (5).

(5) $$\Lambda =\left|\begin{array}{ccccc} p_{11}(\Delta t) & q\cdot p_{12}(\Delta t) & \left(1-q)\cdot p_{12}(\Delta t\right) & \cdots & p_{1N}(\Delta t)\\ p_{21}(\Delta t) & p_{22}(\Delta t) & p_{23}(\Delta t) & \cdots & p_{2N}(\Delta t)\\ \cdots & \cdots & \cdots & \cdots & \cdots \\ p_{N1}(\Delta t) & p_{N2}(\Delta t) & p_{N3}(\Delta t) & \cdots & p_{NN}(\Delta t) \end{array}\right|$$

where the sum of each row of the matrices in Equations (4) and (5) is equal to 1. Therefore, the elements on the main diagonal of the matrices satisfy Equation (6).

(6) $$p_{ii}(t,t+\Delta t)=1-\sum_{j=1}^{N}p_{ij}(t,t+\Delta t),i\neq j$$

The probability row vector *Q*(*t*), which represents the probability of the system in each state at time *t*, can be calculated by Equation (7).

(7) $$Q(t)=Q(0)\Lambda^{t/\Delta t}$$

where Q(0) is the probability row vector of the system in each state at the initial time.

#### 2.2.2. Reliability Assessment Based on the Continuous-Time Homogeneous Markov Process

Similar to the STPM of the discrete-time Markov model, a state transition rate matrix (STRM) of the continuous-time homogeneous Markov process is constructed when the state of the system is observed in continuous time. The STRM of the system reliability model based on a continuous-time homogeneous Markov process, the counterpart of Equation (4), can be expressed using Equation (8).

(8) $$\Lambda^{*}=\left|\begin{array}{cccc} -\sum_{j=2}^{N}\alpha_{1j} & \alpha_{12} & \cdots & \alpha_{1N}\\ \alpha_{21} & -\sum_{\begin{array}{c} j=1\\ j\neq 2 \end{array}}^{N}\alpha_{2j} & \cdots & \alpha_{2N}\\ \cdots & \cdots & \cdots & \cdots \\ \alpha_{N1} & \alpha_{N2} & \cdots & -\sum_{j=1}^{N-1}\alpha_{Nj} \end{array}\right|$$

The probability row vector Q(t) of the system in each state at time *t* satisfies:

(9) $$\frac{dQ(t)}{dt}=Q(t)\Lambda^{*}$$

Given Q(0), the probability row vector Q(t) can be solved by applying the Laplace transform to Equation (9).

The scale of either the STPM of a discrete-time homogeneous Markov model or the STRM of a continuous-time homogeneous Markov model increases exponentially with the number of system components. As the scale of the system increases, it becomes increasingly challenging to model it using the Markov method, which is referred to as the “curse of dimensionality” problem.

## 3. Proposed Method

To address the “curse of dimensionality” problem that arises when applying the Markov method to the reliability assessment for complex systems, a reliability assessment for complex systems based on non-homogenous Markov processes is proposed. It is thoroughly discussed in this section.

### 3.1. Reliability Assessment Based on the Non-Homogeneous Markov Process

#### 3.1.1. Calculation of the Number and Numbering of System States

Let us assume that the system consists of *n* components. Each component can have a different number of states: $C_{i}$ represents the *i*-th component and $Nc_{i}$ is the number of states of the *i*-th component. The set of states of all components is used to represent the system state, i.e., $\left(Sc_{1},Sc_{2},\cdots ,Sc_{n}\right)$, in which $Sc_{i}$ represents the state of component $C_{i}$; the value range of $Sc_{i}$ is $1\leq Sc_{i}\leq Nc_{i}$; $Sc_{i}=1$ represents the new state of the component; and the following values represent the degraded state and the failure state, respectively. Then, the number of system states can be calculated as follows:

(10) $$N=\prod_{i=1}^{n}Nc_{i}$$

Equation (11) is used to number the system states. The numbering of each system state $\left(Sc_{1},Sc_{2},\cdots ,Sc_{n}\right)$ ranges in $1\;\leq \;L(Sc_{1},Sc_{2},\cdots ,Sc_{n})\;\leq N$ and is unique.

(11) $$L(Sc_{1},Sc_{2},\cdots ,Sc_{n})=\sum_{j=1}^{n-1}\left[(\prod_{i=1}^{n}Sc_{i})(Sc_{j}-1)\right]$$

#### 3.1.2. Solution of the Possible Transitions of System States

Assumption: In the non-homogeneous Markov model, the mission time $T_{m}$ is divided into a finite number of discrete time points with equal intervals, and the system state is observed at the discrete time points. The interval between two adjacent time points Δt is sufficiently small that, at most, one component has a state transition within a Δt.

To find the possible transitions between system states, the traversal method compares *N* states of the system pairwise. Because it is assumed that, at most, one component has a state transition within a Δt, it is possible for two system states to transition to each other only when there is only one element different between the two system states.

As an illustration, if we compare system states $N_{i}$ and $N_{j}$, only the *k*-th element, i.e., the state of component $C_{k}$, differs. The state of component $C_{k}$ is state *a* and state *b* in system states $N_{i}$ and $N_{j}$, respectively, $a,b\in [1,N_{ck}]$. Then, state $N_{i}$ and state $N_{j}$ can be transitioned to each other within a Δt. This implies that if the system is in state $N_{i}$ at time *t*, it is possible that the system may transition from state $N_{i}$ to state $N_{j}$ in the time interval (t,t+Δt) due to the transition of component $C_{k}$ from state *a* to state *b* and the absence of state transitions in other components in the interval (t,t+Δt). The probability of this event can be calculated as follows:

(12) $$p_{ij}(t,t+\Delta t)=\{1-\exp[-\int_{t}^{t+\Delta t}\lambda_{k,a,b}(t)dt]\}\cdot \prod_{m\neq k}\left\{\exp[-\int_{t}^{t+\Delta t}\lambda_{m}(t)dt]\right\}$$

where $\lambda_{k,a,b}$ denotes the transition rate of the component $C_{k}$ from state *a* and state *b*, and $\lambda_{m}$ denotes the transition rate of the component $C_{m}$ from the current state to other states.

If the Δt is sufficiently small, $p_{ij}(t,t+\Delta t)$ can be approximated as follows:

(13) $$p_{ij}(t,t+\Delta t)=1-\exp[-\lambda_{k,a,b}(t)\cdot \Delta t]$$

It is also possible that, as component $C_{k}$ transitions from state *b* to state *a* in the interval (t,t+Δt) and the other components remain in the states they are in at time *t*, the system will transition from state $N_{j}$ to state $N_{i}$ before time (t+Δt). The probability of this event can be approximated as follows:

(14) $$p_{ij}(t,t+\Delta t)=1-\exp[-\lambda_{k,b,a}(t)\cdot \Delta t]$$

#### 3.1.3. Generation of Dynamic STPM

All possible system state transition probabilities obtained in Section 3.1.2 are incorporated into the STPM (Equation (15)). The remaining elements of the non-main diagonal are set to 0, indicating that the corresponding system state transitions are assumed not to occur in the interval (t,t+Δt). The values of the elements of the main diagonal are calculated using Equation (16). The dynamic STPM of the system Λ(t+Δt) is obtained.

(15) $$\Lambda (t+\Delta t)=\left[\begin{array}{cccc} p_{11}(t,t+\Delta t) & p_{12}(t,t+\Delta t) & \cdots & p_{1N}(t,t+\Delta t)\\ p_{21}(t,t+\Delta t) & p_{22}(t,t+\Delta t) & \cdots & p_{2N}(t,t+\Delta t)\\ \cdots & \cdots & \cdots & \cdots \\ p_{N1}(t,t+\Delta t) & p_{N2}(t,t+\Delta t) & \cdots & p_{NN}(t,t+\Delta t) \end{array}\right]$$

(16) $$p_{ii}(t,t+\Delta t)=1-\sum_{j=1}^{N}p_{ij}(t,t+\Delta t),i\neq j$$

#### 3.1.4. System Unavailability Calculation

Given that the probability of the system being in state *i* at the initial time is q(i,0), the probability vector of the system’s initial state of Q(0) can be represented by Equation (17).

(17) Q(0)=[q(1,0),q(2,0),⋯,q(i,0),⋯,q(N,0)]

The dynamic STPM for the first time step Λ(Δt) can be obtained in accordance with Equations (15) and (16), and the probability row vector of the system state at the first time point Q(Δt) can be obtained by substituting Λ(Δt) and Equation (17) into Equation (18). The dynamic STPM is updated at each time step, and the probability row vector of the system being in each state at each time point on the discrete time axis is iteratively calculated.

(18) Q(t+Δt)=[q(1,t+Δt),q(2,t+Δt),⋯,q(N,t+Δt)]=Q(t)Λ(t+Δt)

Given the system failure state set *S_F_* in the system state set *S*, the unavailability of the system at time *t* is equal to the sum of the probabilities of the system being in all failure states at time *t*, as shown in Equation (19).

(19) $$\bar{A}(t)=\prod_{i\in S_{F}}q(i,t),S_{F}\in S$$

### 3.2. Reliability Assessment for Complex Systems

**Figure 1:**
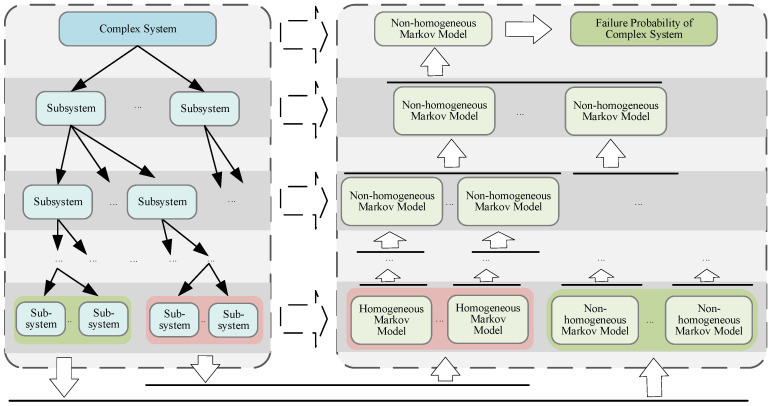
Schematic diagram of reliability assessment for complex systems based on non-homogeneous Markov processes.

**Figure 2:**
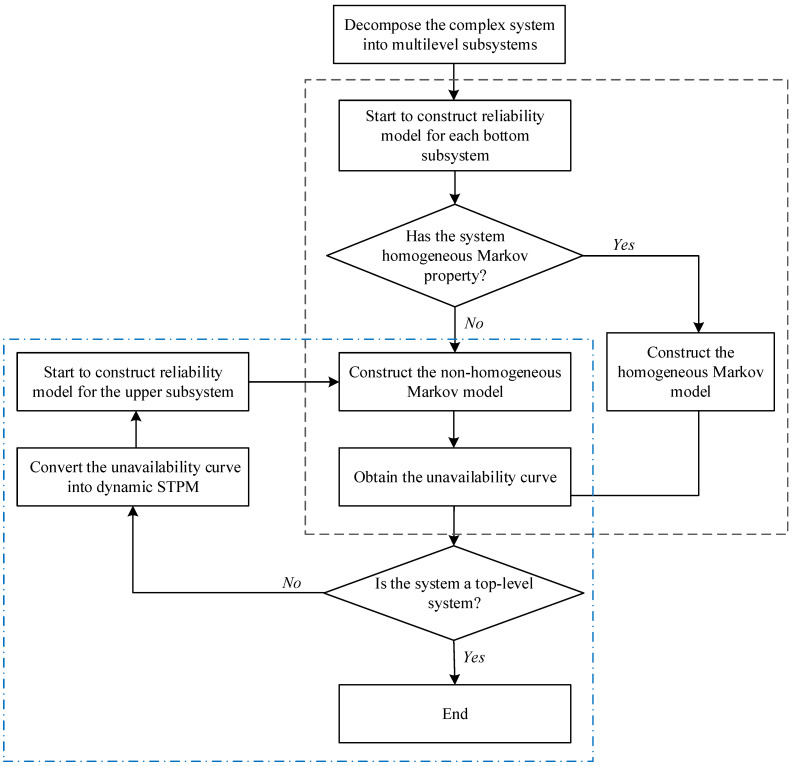
Flow chart of the implementation of a reliability assessment for complex systems based on the proposed method.

As shown in Figure 1, the basic idea of the reliability assessment method for complex systems based on non-homogeneous Markov processes proposed in this paper is as follows: the complex system is divided into multi-level subsystems according to the system function, and the scales of subsystems are controlled. Homogeneous Markov models or non-homogeneous Markov models are constructed for each subsystem/system from bottom to top. In the case that the bottom-level subsystems have homogeneous Markov properties, the reliability models based on the discrete-time or continuous-time Markov process can be constructed as described in Section 2.2; otherwise, if the subsystems only have non-homogeneous Markov properties, the non-homogeneous Markov reliability models are constructed using the method proposed in Section 3.1. The unavailability curves of the bottom-level subsystems are obtained from the models and then are converted into their corresponding 2×2 dynamic STPMs. The 2×2 dynamic STPMs serve as the inputs for developing a non-homogeneous Markov reliability model of the upper-level subsystem. Finally, the model of the top-level system is completed, and the unavailability of the system is obtained. The flow chart of the implementation of the reliability assessment for complex systems based on the proposed method is illustrated in Figure 2. The gray dashed box represents the reliability modeling process of the bottom-level subsystem, while the blue dashed box depicts the reliability modeling process of the non-bottom-level subsystems or the top-level system.

A significant challenge in the aforementioned process is the conversion of the unavailability curves of the lower-level subsystems into the inputs of the non-homogeneous Markov reliability model of the upper-level subsystem. To address this issue, a novel algorithm is presented for converting the unavailability curve of a subsystem into its corresponding dynamic STPM, as detailed below.

**Figure 3:**
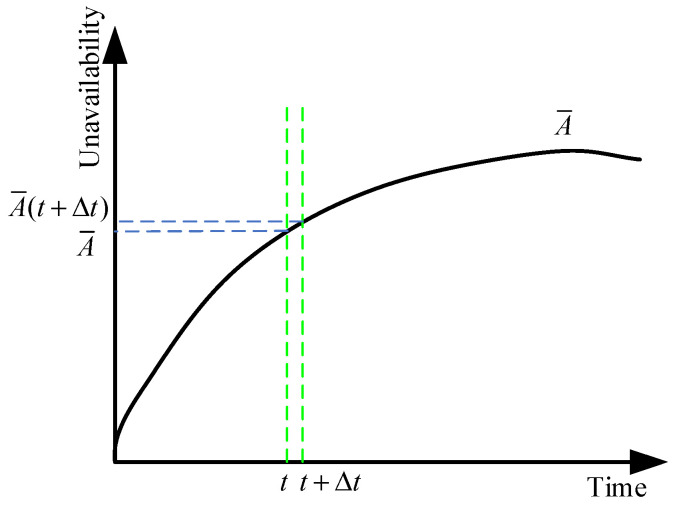
Diagram of the unavailability curve of a subsystem.

Assumption: the subsystems have only two states: normal operation state and failure state (denoted as state 1 and state 2, respectively). Figure 3 is a diagram of a subsystem unavailability curve obtained from the homogeneous Markov model or non-homogeneous Markov model of a subsystem. Based on the aforementioned assumption, if the contribution of failure behavior to the unavailability of the subsystem is greater than that of the repair and maintenance behavior, the unavailability will increase; otherwise, it will decrease. The 2×2 dynamic STPM of a subsystem can be expressed by Equation (20).

(20) $$\Lambda_{s}=\left[\begin{array}{cc} 1-v_{12}(t,t+\Delta t) & v_{12}(t,t+\Delta t)\\ v_{21}(t,t+\Delta t) & 1-v_{21}(t,t+\Delta t) \end{array}\right]$$

It is further assumed that the variation of the subsystem unavailability within a time interval Δt is contributed only by the failure behavior or the repair behavior. The subsystem unavailability curves are converted into their corresponding 2×2 dynamic STPMs according to the following three cases:

(1) If the unavailability of the subsystem increases in (t,t+Δt), it is assumed that the subsystem has no repair behavior in this process, and the increase in unavailability is contributed by the failure behavior. The probability of the subsystem transitioning from the normal operation state to the failure state $v_{12}(t,t+\Delta t)$ is calculated as follows:

(21) $$v_{12}(t,t+\Delta t)=\frac{\bar{A}(t+\Delta t)-\bar{A}(t)}{1-\bar{A}(t)}=\frac{\Delta \bar{A}(t,t+\Delta t)}{1-\bar{A}(t)}$$

The probability of the subsystem transitioning from the failure state to the normal operation state $v_{21}(t,t+\Delta t)$ is 0.

(22) $$v_{21}(t,t+\Delta t)=0$$

(2) If the unavailability of the subsystem decreases in (t,t+Δt), it is assumed that the subsystem has repair behavior but no failure behavior in this process. $v_{12}(t,t+\Delta t)$ and $v_{21}(t,t+\Delta t)$ are calculated as follows:

(23) $$v_{12}(t,t+\Delta t)=0$$

(24) $$v_{21}(t,t+\Delta t)=\frac{A(t+\Delta t)-A(t)}{\bar{A}(t)}=\frac{-\Delta \bar{A}(t,t+\Delta t)}{\bar{A}(t)}$$

(3) If the unavailability of the subsystem remains unchanged in (t,t+Δt), $v_{12}(t,t+\Delta t)$ and $v_{21}(t,t+\Delta t)$ are equal to 0.

(25) $$v_{12}(t,t+\Delta t)=v_{21}(t,t+\Delta t)=0$$

In constructing the non-homogeneous Markov model of the upper-level system, the 2×2 dynamic STPMs of the lower-level systems are taken as inputs to update the dynamic STPM of the upper-level system at each time step. Each element in the dynamic STPM of the upper-level system that represents the state transition of the upper-level system caused by the lower-level subsystems is replaced with its corresponding state transition probability from the 2×2 dynamic STPM of the lower-level systems. The model is constructed according to the modeling steps in Section 3.1, and the unavailability curve of the upper-level system is obtained.

## 4. Case Study

### 4.1. System Description

**Figure 4:**
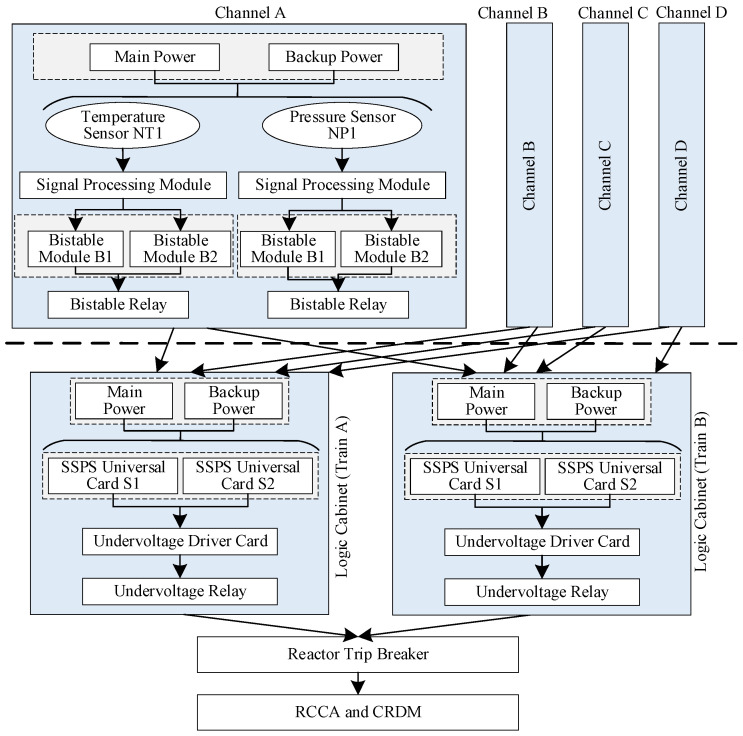
Diagram of the RPS structure.

A reactor protection system (RPS) of a nuclear power plant was taken as an application [28,29]. The system consisted of four main parts: an instrumentation rack, a logic cabinet, a reactor trip breaker, and a control rod module (including the rod control cluster assemblies (RCCA) and the control rod drive mechanisms (CRDM)). The instrumentation rack was equipped with a total of four independent signal channels (A, B, C, D). The signals from the instrumentation rack were transmitted to the two trains of the logic cabinet after a 2-out-of-4 selection. A signal channel was composed of a temperature sensor, a pressure sensor, two signal processing modules, four bistable modules, two bistable relays, and a power module (including a main power and a backup power). A train is composed of two SSPS universal cards, an undervoltage driver card, an undervoltage relay, and a power module. As long as there is an output signal from either train, the reactor shutdown procedure is initiated. The system structure is illustrated in Figure 4.

The failure parameters for each component of the RPS system are presented in Table 1, with the corresponding failure data cited in the references [28,29,30]. This paper considers the degradation of bistable relays and refers to the reliability model of aerospace relays described in reference [31]. The failure process of the relays was modeled using a lognormal distribution, and its reliability can be expressed using Equation (26).

(26) $$R(t)=1-\phi (\frac{\ln t-11.89}{0.63})$$

In order to simplify the study, only two shutdown signals, namely, the overpower ∆T and pressurizer high pressure trip signals, were considered in this case. The event of system failure was defined as the RPS system failing to shut down.

### 4.2. Reliability Modeling

As illustrated in Figure 4, the RPS system was divided into six Level 1 subsystems based on the system function, as shown in the graphic boxes with a solid line and powder blue coloring. There are four signal channel subsystems and two train subsystems. Each signal channel subsystem was further divided into two Level 2 subsystems: a channel power subsystem and a bistable module subsystem, along with six other components. Each train subsystem was further divided into two Level 2 subsystems: a train power subsystem, a solid-state protection subsystem, and two additional components. The Level 2 subsystems, which represent the bottom-level subsystems in this case, are delineated by graphic boxes with a dashed line and gray coloring.

The reliability modeling of the RPS system based on the proposed method was completed in three stages. First, the homogeneous Markov reliability models were constructed for each Level 2 subsystem in order to obtain the unreliability curves of the subsystems from the corresponding models. Second, the non-homogeneous Markov reliability models were constructed for each Level 1 subsystem, resulting in the unreliability curves of each subsystem. Finally, a non-homogeneous Markov reliability model of the RPS system was constructed, and the unreliability curve of the entire system was obtained. The time interval Δt for all models was set to 1 h, and the mission time was set to 5000 h.

**Figure 5:**
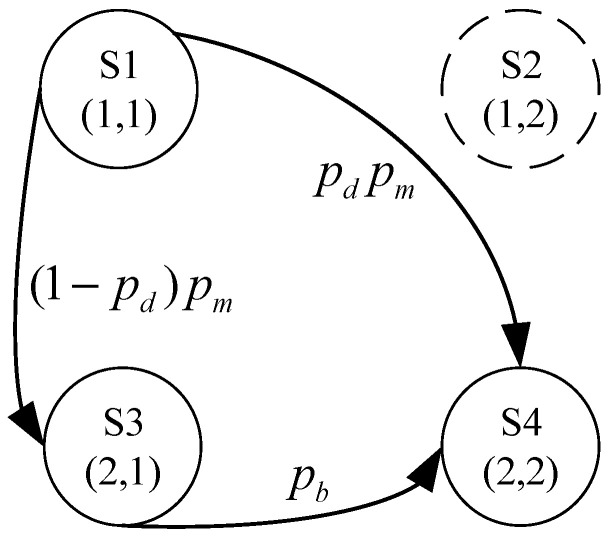
State transition diagram of the power channel subsystem.

A channel power subsystem (power module) was taken as an example to illustrate the process of the Markov reliability modeling of the bottom-level subsystems. The channel power subsystem consisted of a main power and a backup power. It was assumed that the backup power would automatically start immediately when the main power failed. The failure probability of starting the backup power is denoted as $P_{d}$. The failure probabilities of the main power and the backup power within a time interval Δt are denoted as $P_{m}$ and $P_{b}$, respectively. The number of system states and the numbering of each system state were calculated using Equations (10) and (11). The state transition diagram of the channel power subsystem is shown in Figure 5.

As shown in Figure 5, the STPM of the channel power subsystem was formulated as shown in Equation (27).

(27) $$\Lambda =\left[\begin{array}{cccc} 1-p_{m} & 0 & (1-p_{d})p_{m} & p_{d}p_{m}\\ 0 & 1 & 0 & 0\\ 0 & 0 & 1-p_{b} & p_{b}\\ 0 & 0 & 0 & 1 \end{array}\right]$$

The probability vector of the system’s initial state of Q(0) and Equation (27) were incorporated into Equations (7) and (19), thereby yielding the unreliability curve of the channel power subsystem. The unreliability curve was converted into its corresponding 2×2 dynamic STPM according to Section 3.2. In a similar manner, the 2×2 dynamic STPMs of the other bottom-level subsystems were derived from their reliability models. They are taken as inputs of the non-homogeneous Markov reliability models of their upper-level systems.

**Figure 6:**
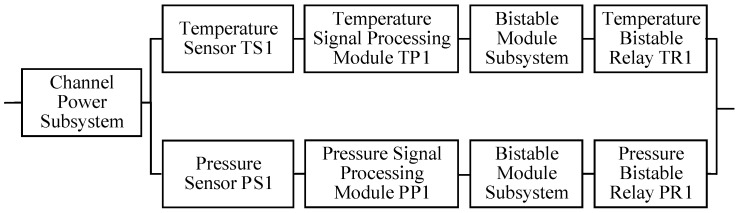
Reliability block diagram of a signal channel subsystem.

A signal channel subsystem served as an illustrative example of the Markov reliability modeling of the Level 1 subsystems. The reliability block diagram, presented in Figure 6, illustrates the reliability function relationships between the signal channel subsystem and the components that comprise the subsystem. The unreliability curve of the bistable relay was calculated based on the reliability distribution in Equation (26), which was converted into its corresponding 2×2 dynamic STPM. The 2×2 dynamic STPMs of the bistable relay, the channel power subsystem, and the bistable module subsystem, along with the failure rates of the temperature sensor and signal processing module, were employed as inputs in the construction of a non-homogeneous Markov reliability model for the single channel subsystem using the method proposed in Section 3.1. The unreliability curve of the signal channel subsystem was derived using Equations (18) and (19) and then was converted into its corresponding 2×2 dynamic STPM. Similarly, a non-homogeneous Markov reliability model of the RPS system was constructed and resolved based on the 2×2 dynamic STPMs of the four signal channel subsystems and two train subsystems.

If the reliability model of the RPS system is constructed directly by the Markov method, the scale of the STPM of the model is $2^{60}\times 2^{60}$. In this case, the reliability assessment of the RPS system was completed by the proposed method, which constructs three homogeneous Markov models and three non-homogeneous Markov models from bottom to top. The scales of the STPMs of the six models are 4×4, 4×4, 4×4, 16×16, 512×512, and 64×64, respectively. The scale and complexity of the model have been significantly simplified, and the problem of “curse of dimensionality” has been effectively addressed.

### 4.3. Model Verification and Result Analysis

#### 4.3.1. Model Verification and System Unreliability Calculation

Two models constructed using Markov/theoretical analysis and sequential Monte Carlo simulation (MCS) were employed to verify the effectiveness and accuracy of the proposed method. In the Markov/theoretical analysis model, the Level 1 subsystems were modeled using a discrete-homogeneous Markov method, and the outcomes were taken as inputs for modeling the RPS system by theoretical analysis based on the reliability theory. The degradation of bistable relays was not considered, and their failure rates were set to 5.81 × 10^−6^/h in this model [31]. In the sequential MCS model, a large number of system operation sequences were simulated, and the reliability parameters of the system, such as unreliability, can be obtained through the statistics and analysis of the sequences. The more sequences simulated, the closer the estimated parameters are to the real values [2]. The key parameters in the sequential MCS model were set as follows: the mission time was set to 5000 h, the number of simulations was 1,000,000, and the time interval was set to 1 h.

**Figure 7:**
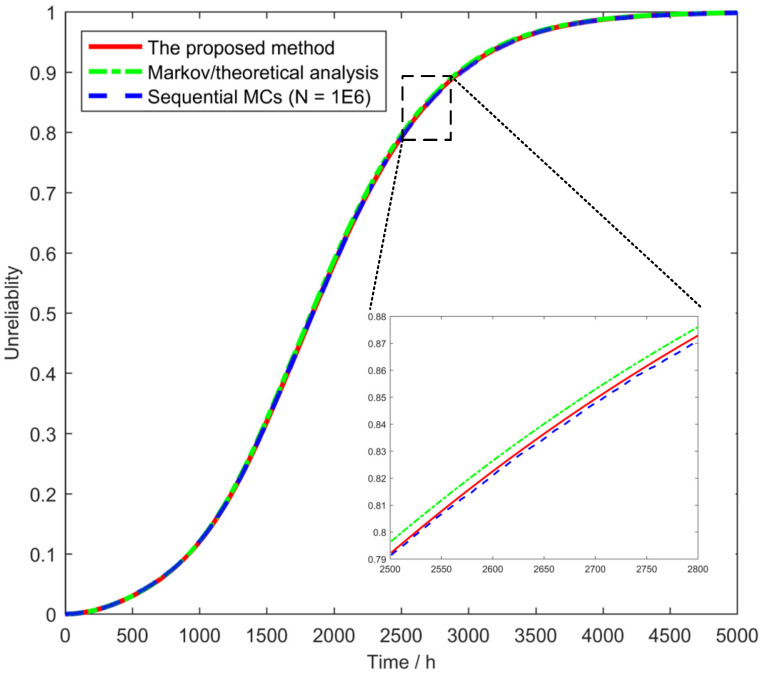
Unreliability curves of the RPS system.

Three reliability assessment models of the RPS system were constructed based on the proposed method, Markov/theoretical analysis and sequential MCS, respectively. Figure 7 illustrates the unreliability curves of the RPS system calculated by the three models. It can be observed that the results of the three models are essentially identical, which indicates that the proposed method is an effective means of modeling the reliability of the RPS system, with accurate calculation results.

The discrepancy between the curves from the proposed model and the contrast models was quantified using a series of quantitative metrics, such as the mean squared error (MSE), the root mean square error (RMSE), the mean absolute error (MAE), and the R-Squared. These quantitative metrics are calculated and presented in Table 2. The results demonstrate that the proposed model exhibits greater consistency with the sequential MCS model. This is due to the fact that both models considered the degradation process of bistable relays, whereas the Markov/theoretical analysis model did not.

#### 4.3.2. Importance Analysis

In order to evaluate the importance of the components, two importance metrics, Risk Achievement Worth (RAW) and Risk Reduction Worth (RRW), are introduced in this paper [32]. The RAW value of a component is defined as the ratio of the unreliability of the system calculated without credit for successful performance of the component ${\bar{R}}_{i}^{+}$ to the baseline unreliability value $\bar{R}$ as shown in Equation (28), which reflects the effect of the failure of the component on the current system risk.

(28) $$\mathrm{RAW}=\frac{{\bar{R}}_{i}^{+}}{\bar{R}}$$

The RRW value of a component is defined as the ratio of the baseline unreliability value $\bar{R}$ to the risk recalculated with credit for successful performance of the component ${\bar{R}}_{i}^{-}$ as shown in Equation (29), which reflects the degree of system risk reduction by the component’s perfect operation.

(29) $$\mathrm{RAW}=\frac{\bar{R}}{{\bar{R}}_{i}^{-}}$$

**Figure 8:**
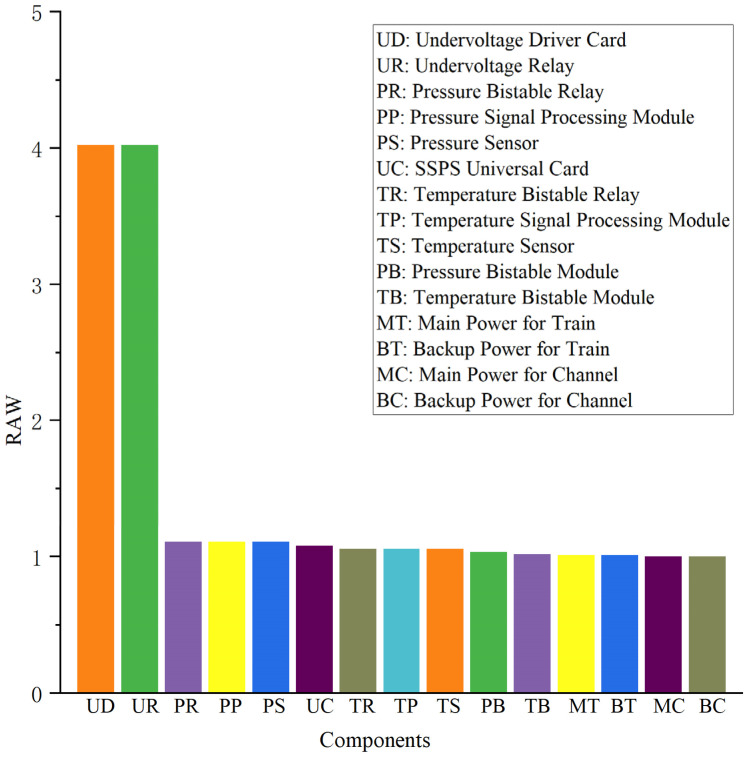
RAW values of each component of the RPS.

**Figure 9:**
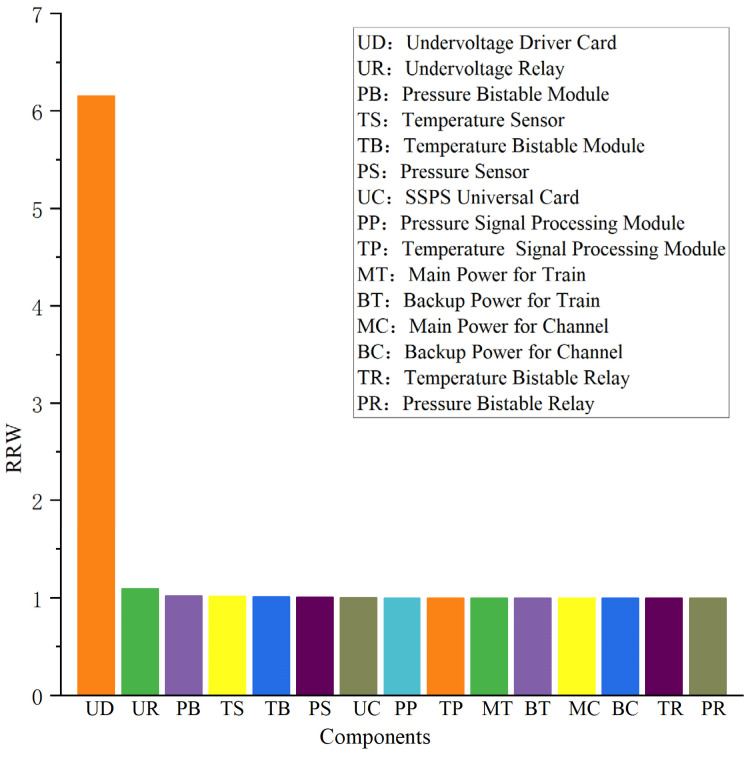
RRW values of each component of the RPS.

In this case, the mission time is set to 720 h (1 month), and the RAW and RRW values of each component of the RPS system within the mission time are calculated, and the results are shown in Figure 8 and Figure 9, respectively.

As illustrated in Figure 7, the two components, the undervoltage driver card (UD) and the undervoltage relay (UR), have the largest RAW values of 4.02. This indicates that the failure of either one of them leads to an increase in the probability of system failure before 720 h by a factor of 4.02. Meanwhile, as can be seen from Figure 8, the RRW value of the undervoltage driver card (UD) is 6.16, which means that the probability of system failure before 720 h can be reduced to 0.16 times of the baseline one if this component does not fail during the mission time. Therefore, it is of great significance for the safe and reliable operation of the RPS system to strengthen the monitoring of the operating status of the undervoltage driver card (UD) and the undervoltage relay (UR) and to improve the reliability requirements of the undervoltage driver card (UD).

## 5. Conclusions

In order to solve the “curse of dimensionality” problem associated with the application of the Markov method in reliability assessments for complex systems, this paper develops a reliability assessment method based on non-homogeneous Markov processes and conducts a case study of a reliability assessment of the RPS system based on the proposed method. The following conclusions are drawn:

(1) A reliability assessment method for complex systems based on non-homogeneous Markov processes is proposed. The results of the case study show that the proposed method effectively solves the “curse of dimensionality” problem.
(2) The degradation process of a component or system can be represented by non-exponential distributions in the proposed method, which enhances the accuracy of the model.
(3) The proposed method is applied to the reliability assessment modeling of the RPS system, and the Markov/theoretical analysis model and sequential MCS model are used as the contrast models. The MSEs, RMSEs, MAEs and R-Squareds of the unreliability curves of the RPS system from the proposed model and the two contrast models are calculated. The results verify the effectiveness and accuracy of the proposed method.
(4) Based on the proposed model, the RAW and RRW values of each component of the RPS system with a mission time of 720 h are calculated. The analysis results show that the undervoltage driver card and the undervoltage relay have the largest RAW values, and the undervoltage driver card has the largest RRW value. Strengthening the monitoring of the undervoltage driver card and the undervoltage relay and increasing the reliability requirements of the undervoltage driver card will be of great significance for improving the safety and reliability of the whole system.

Furthermore, it should be noted that the proposed method is subject to certain limitations. The conversion of the outcomes of the lower-level subsystem models into inputs for the upper-level subsystem model is based on the assumption that subsystems have only two states: a normal operation state and a failure state. However, this may not be the case in practice. Therefore, our subsequent research will focus on developing a solution to address this issue.

## Figures and Tables

**Table 1:** Failure parameters of each component of the RPS.

No.	Component Name	Failure Rate/ Probability
1	Temperature Sensor	4.00 × 10^−4^/h
2	Pressure Sensor	1.20 × 10^−4^/h
3	Signal Processing Module	8.20 × 10^−6^/h
4	Bistable Module	7.40 × 10^−4^/h
5	Main Power	4.00 × 10^−6^/h
6	Backup Power	5.00 × 10^−6^/h
7	SPS Universal Card	3.80 × 10^−5^/h
8	Undervoltage Driver Card	3.40 × 10^−4^/h
9	Undervoltage Relay	3.90 × 10^−5^/h
10	Demand of Backup Power	0.01/D

**Table 2:** The metrics for measuring the difference of curves from the three models.

Models	MSE	RMSE	MAE	R-Squared
Proposed method and Markov/theoretical analysis	7.05 × 10^−6^	0.002655	0.001903	0.999952
Proposed method and sequential MCS	7.60 × 10^−7^	0.000872	0.000653	0.999995

## Data Availability

The raw data are contained within the article.

## References

[1] International Atomic Energy Agency (2010). Development and Application of Level 1 Probabilistic Safety Assessment for Nuclear Power Plants.

[2] Pan X., Di Maio F., Zio E. A Benchmark of Dynamic Reliability Methods for Probabilistic Safety Assessment. Proceedings of the 2017 2nd International Conference on System Reliability and Safety (ICSRS).

[3] Wiltbank N.E., Palmer C.J. (2021). Dynamic PRA Prospects for the Nuclear Industry. Front. Energy Res..

[4] Kirschenbaum J., Bucci P., Stovsky M., Mandelli D., Arndt S.A. (2009). A Benchmark System for Comparing Reliability Modeling Approaches for Digital Instrumentation and Control Systems. Nucl. Technol..

[5] Raveendran A., Renjith V.R., Madhu G. (2022). A Comprehensive Review on Dynamic Risk Analysis Methodologies. J. Loss Prev. Proc..

[6] Wu Y. (2015). Development of Reliability and Probabilistic Safety Assessment Program RiskA. Ann. Nucl. Energy.

[7] Park J.W., Lee S.J. (2022). Simulation Optimization Framework for Dynamic Probabilistic Safety Assessment. Reliab. Eng. Syst. Safe.

[8] Aldemir T. (2013). A Survey of Dynamic Methodologies for Probabilistic Safety Assessment of Nuclear Power plants. Ann. Nucl. Energy.

[9] Aldernir T., Miller D., Stovsky M., Kirschenbaurr J., Bucci P., Fentiman A., Mangan L. (2006). Current State of Reliability Modeling Methodologies for Digital Systems and Their Acceptance Criteria for Nuclear Power Plant Assessments (NUREG/CR-6901).

[10] Pan X.L., Wang J.Q., Yuan R., Wang F., Lin H.Q., Hu L.Q., Wang J. (2017). Biasing Transition Rate Method Based on Direct MC Simulation for Probabilistic Safety Assessment. Nucl. Sci. Tech..

[11] Song C., Kawai R. (2023). Monte Carlo and Variance Reduction Methods for Structural Reliability Analysis: A Comprehensive Review. Probabilistic Eng. Mech..

[12] Yilmaz S., Gueltekin O.E. (2023). Reliability Analysis of Repairable Multistate Phased Mission Systems with Markov Approach Based on States. Eng. Comput..

[13] Lyu H., Qu H., Xie H., Zhang Y., Pecht M. (2023). Reliability Analysis of The Multi-state System with Nonlinear Degradation Model under Markov Environment. Reliab. Eng. Syst. Safe.

[14] Hellmich M., Berg H.-P. (2015). Markov Analysis of Redundant Standby Safety Systems under Periodic Surveillance Testing. Reliab. Eng. Syst. Safe.

[15] Rajeevan A.K., Shouri P.V., Nair U. (2018). Markov Modeling and Reliability Allocation in Wind Turbine for Availability Enhancement. Life Cycle Reliab. Saf. Eng..

[16] Bessman J.C. Non-Homogeneous Markov Models and Their Application in Reliability. Proceedings of the 2020 Annual Reliability and Maintainability Symposium (RAMS).

[17] Chen G., Gaebler J.D., Peng M., Sun C., Ye Y. (2021). An Adaptive State Aggregation Algorithm for Markov Decision Processes. arXiv.

[18] Jia C. (2016). Simplification of Irreversible Markov Chains by removal of States with Fast Leaving Rates. J. Theor. Biol..

[19] Liang Q., Yang Y., Zhang H., Peng C., Lu J. (2022). Analysis of Simplification in Markov State-based Models for Reliability Assessment of Complex Safety Systems. Reliab. Eng. Syst. Safe.

[20] Zuo W., Li K. Three-State Markov Chain Based Reliability Analysis of Complex Traction Power Supply Systems. Proceedings of the 2021 5th International Conference on System Reliability and Safety (ICSRS).

[21] Durga Rao K., Gopika V., Sanyasi Rao V.V.S., Kushwaha H.S., Verma A.K., Srividya A. (2009). Dynamic Fault Tree analysis Using Monte Carlo Simulation in Probabilistic Safety Assessment. Reliab. Eng. Syst. Safe.

[22] Jiang C., He Z., Li F., Xie F., Zheng L., Yang J., Yang M. (2023). A Hybrid Computing Framework for Risk-oriented Reliability Analysis in Dynamic PSA Context: A Case Study. Qual. Reliab. Eng. Int..

[23] Abdulmunem A.H., Al-Khafaji Z. Using Markov Models and Fault Tree for Finding the Reliability of Some Engineering Problems. Proceedings of the 2023 6th International Conference on Engineering Technology and its Applications (IICETA).

[24] Son K.S., Seong S.H., Jang G.S., Kang H.G. (2020). Periodic Surveillance Test Strategies to Effectively Enhance the Availability of Safety-critical Systems in NPPs Using the Multi-state Based Availability Model. Ann. Nucl. Energy.

[25] Ullah G., Bruno W.J., Pearson J.E. (2012). Simplification of Reversible Markov Chains by Removal of States with Low Equilibrium Occupancy. J. Theor. Biol..

[26] Jia C. (2017). Simplification of Markov Chains with Infinite State Space and the Mathematical Theory of Random Gene Expression Bursts. Phys. Rev. E.

[27] (2000). Functional Safety of Electrical/Electronic/Programmable Electronic Safety Related Systems.

[28] Li X., Ge D., Lin Z., Wang S., Wang J. (2021). Application of Dynamic Fault Tree in Reliability Assessment of Reactor Protection System. Nucl. Tech..

[29] Eide S.A., Calley M.B., Gentillon C.A., Wierman T.E., Rasmuson D., Marksberry D. Westinghouse Reactor Protection System Unavailability, 1984–1995. Proceedings of the PSA ‘99.

[30] Fahmy R.A. (2021). Development of Dynamic Fault Tree Model for Reactor Protection System. Process Saf. Prog..

[31] Yu Q. (2012). Rechearch on Reliability Evaluation and Life Test Methods for Sppace Relays. Ph.D. Thesis.

[32] Zio E., Pham H. (2011). Risk Importance Measures. Safety and Risk Modeling and Its Applications.

